# Six New Polyketide Decalin Compounds from Mangrove Endophytic Fungus *Penicillium aurantiogriseum* 328#

**DOI:** 10.3390/md13106306

**Published:** 2015-10-10

**Authors:** Yanhong Ma, Jing Li, Meixiang Huang, Lan Liu, Jun Wang, Yongcheng Lin

**Affiliations:** 1 School of Pharmaceutical Sciences, Sun Yat-sen University, Guangzhou 510006, China; E-Mails: mayh6@mail2.sysu.edu.cn (Y.M.); huangmx7@mail2.sysu.edu.cn (M.H.); 2 School of Marine Sciences, Sun Yat-sen University, Guangzhou 510006, China; E-Mail: lijing356@mail.sysu.edu.cn; 3 Key Laboratory of Functional Molecules from Oceanic Microorganisms (Sun Yat-sen University), Department of Education of Guangdong Province, Guangzhou 510080, China; E-Mail: ceslyc@mail.sysu.edu.cn; 4 South China Sea Bio-Resource Exploitation and Utilization Collaborative Innovation Center, Guangzhou 510006, China; 5 School of Chemistry and Chemical Engineering, Sun Yat-sen University, Guangzhou 510275, China

**Keywords:** mangrove endophytic fungi, *Penicillium aurantiogriseum*, secondary metabolites, polyketide decalin derivative

## Abstract

Six new compounds with polyketide decalin ring, peaurantiogriseols A–F (**1**–**6**), along with two known compounds, aspermytin A (**7**), 1-propanone,3-hydroxy-1-(1,2,4a,5,6,7,8,8a-octahydro-2,5-dihydroxy-1,2,6-trimethyl-1-naphthalenyl) (**8**), were isolated from the fermentation products of mangrove endophytic fungus *Penicillium aurantiogriseum* 328#. Their structures were elucidated based on their structure analysis. The absolute configurations of compounds **1** and **2** were determined by ^1^H NMR analysis of their Mosher esters; the absolute configurations of **3**–**6** were determined by using theoretical calculations of electronic circular dichroism (ECD). Compounds **1**–**8** showed low inhibitory activity against human aldose reductase, no activity of inducing neurite outgrowth, nor antimicrobial activity.

## 1. Introduction

**Figure 1:**
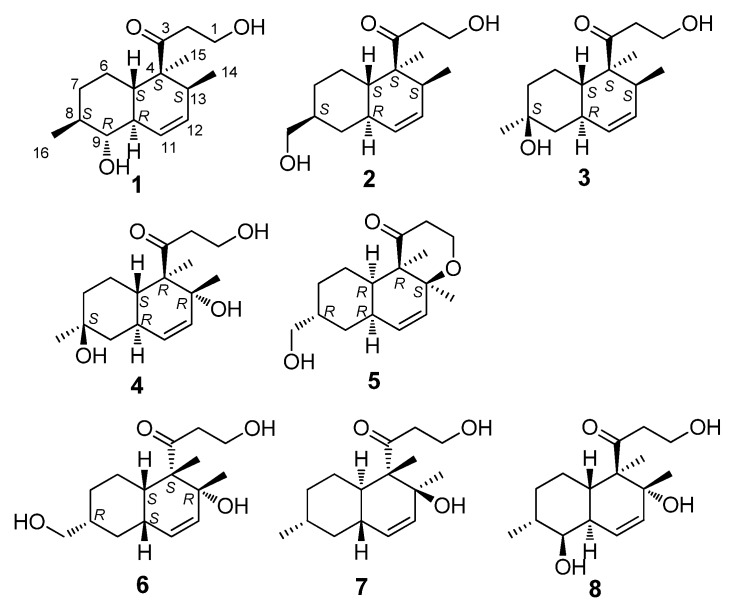
The chemical structures of compounds **1**–**8**.

Mangrove is a specialized marine ecosystem. Mangrove endophytic fungi have drawn a lot of attention for the past few years as a rich source of bioactive and novel compounds [1,2]. In the course of our exploration for the metabolites of endophytic fungi from the mangrove in the South China Sea, numerous new compounds were obtained [3,4,5]. In this study, six new compounds, peaurantiogriseols A–F (**1**–**6**), along with two known compounds, aspermytin A (**7**) and 1-propanone,3-hydroxy-1-(1,2,4a,5,6,7,8,8a-octahydro-2,5-dihydroxy-1,2,6-trimethyl-1-naphthalenyl) (**8**), were isolated from endophytic fungus *Penicillium aurantiogriseum* 328# from the bark of mangrove plant *Hibiscus tiliaceus*. Compounds **1**–**8** (Figure 1) had similar polyketide decalin scaffolds substituted by 3-oxopropanol side chains, and shown low inhibitory activity against 6×His-tagged recombinant human aldose reductase. Here, we report the isolation and structural elucidation of compounds **1**–**8** based on the studies of their NMR, EI-MS, and ECD spectra.

## 2. Results and Discussion

**Figure 2:**
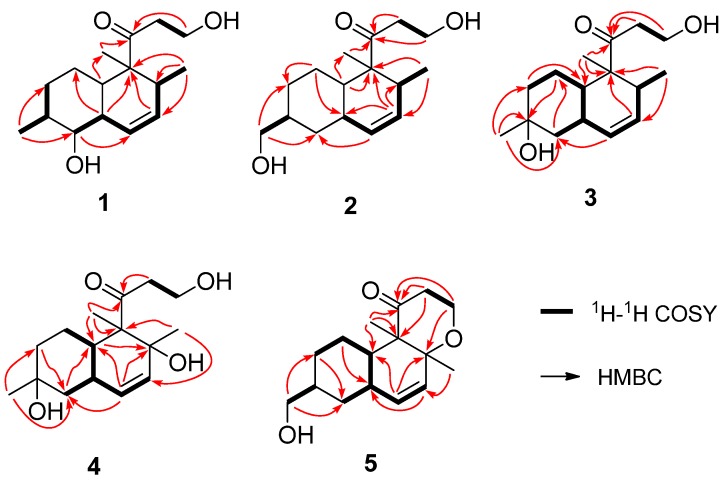
The key ^1^H–^1^H COSY and HMBC correlations of compounds **1**–**5**.

Peaurantiogriseol A (**1**, Figure 1) was obtained as a colorless solid and had a molecular formula of C_16_H_26_O_3_ as determined by HREIMS data (observed *m*/*z* 266.1878 M^+^, calculated 266.1876), requiring 4° of unsaturation. The ^13^C-NMR and DEPT spectra (Table 1) indicated the presence of a carbonyl group (δ 215.4), two olefinic carbons, four *sp*^3^ CH_2_ groups, five *sp*^3^ CH groups, one *sp*^3^ quaternary carbon atom, and three methyl groups. The ^1^H-NMR and ^1^H–^1^H COSY spectra (Table 1 and Figure 2) showed the signals of a 3-oxopropanol system (δ_H_ 3.82/2.64), and a *cis* double bond signals (δ_H_ 5.91 d *J* = 10.6 Hz; 5.58 ddd *J* =10.6, 4.8, 2.4 Hz). The remaining 2° of unsaturation supported a decalin segment in **1**. In the HMBC spectrum (Figure 2), rich correlation data allowed us to unambiguously establish the locations of substituents on the decalin ring. A methyl singlet at δ_H_ 1.19 correlated with C-3 and C-5 respectively, which revealed that the methyl group, with the 3-oxopropanol side chain, was located at C-4 position. A methyl doublet signals at δ_H_ 0.75 (*J* = 8.4 Hz) correlated with C-13 and C-12, and another methyl doublet signals at δ_H_ 1.00 (*J* = 9.6 Hz) correlated with C-9 and C-7, revealing that the two methyl groups were located at C-8 and C-13 positions, respectively. Based on the HMBC correlations of H-11/C-9 and H-12/C-14, the *cis* double bond was easily assigned as C-11 and C-12. One hydroxyl group was identified at C-9 position based on the chemical shift of CH-9 (δ 2.89/79.3) and HMBC correlations.

**Figure 3:**
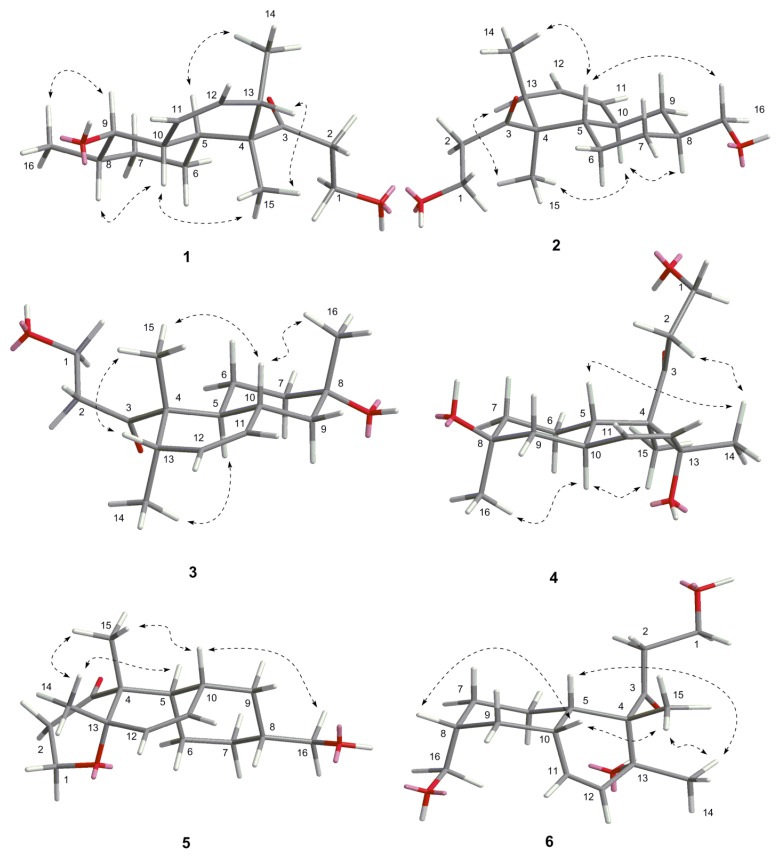
The key NOE correlations of compounds **1**–**6**.

The relative stereochemistry of **1** was established by its NOESY spectrum (Figure 3). The NOE correlations of H-15/H-10, H-15/H-13 and H-14/H-5 confirmed a *trans*-fused decalin ring in **1**. Methyl-15 and OH-9 were oriented on the same side of the decalin ring, and methyl-14 and methyl-16 were oriented on the other side based on the NOE correlations of H-8/H-10 and H-16/H-9.

**Figure 4:**
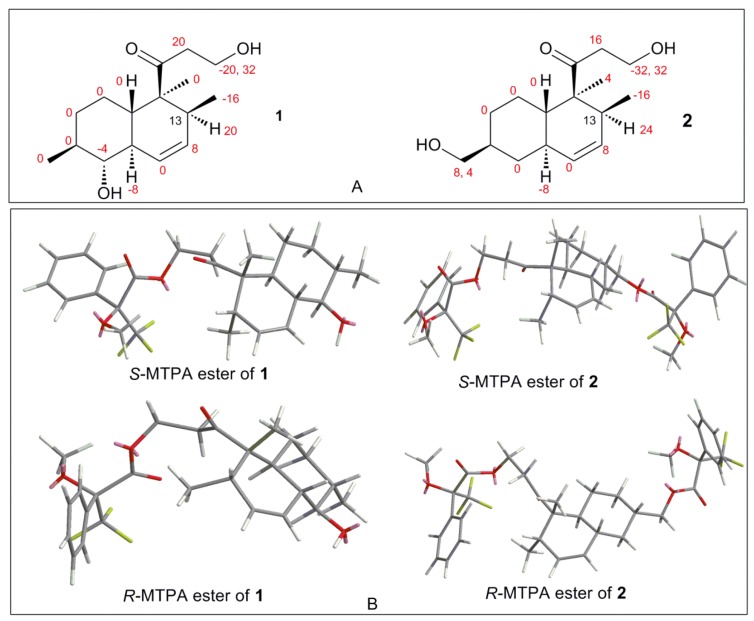
(**A**) Chemical shift difference values (Δδ*^SR^* = δ*^S^* − δ*^R^*, in Hz) of compounds **1**/**2** esterified by *S*/*R*-MTPA-Cl; and (**B**) preferred conformations of *S*/*R*-Mosher esters of **1**/**2**.

**Figure 5:**
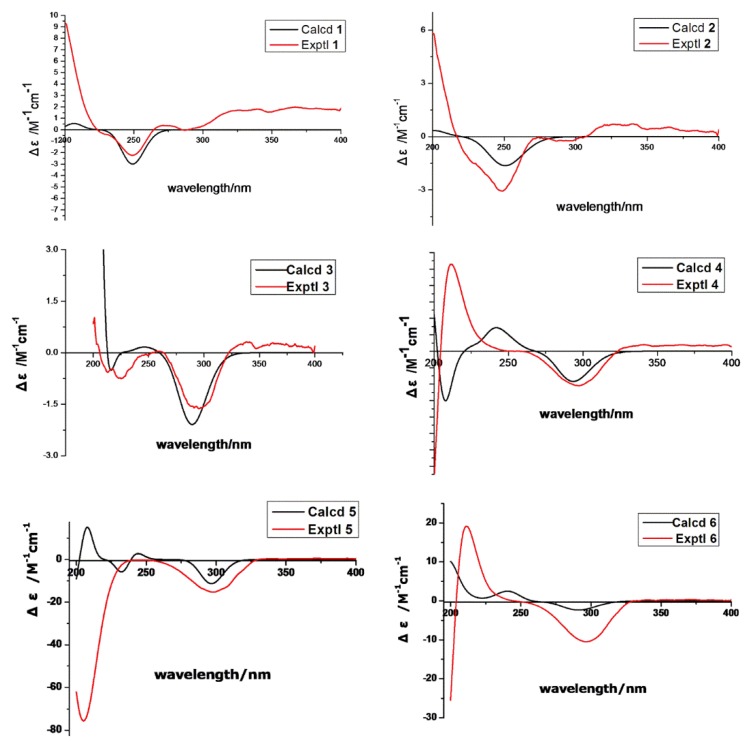
Calculated and experimental ECD spectra of **1**–**6**.

The absolute configuration of **1** was determined by comparison of the ^1^H-NMR spectra of corresponding (*R*) and (*S*)-Mosher esters (Figure 4; Supplementary Materials Table S2). OH-1 of compound **1** was mainly esterified by *S*/*R*-MTPA-Cl based on the larger chemical shift of H-1. There was an esterified C-1 hydroxyl group in Mosher esters of **1**, which were confirmed by their ^19^F NMR spectra that showed 2 CF_3_ signals (Supplementary Materials Figure S8). The preferred conformations of Mosher esters of **1** dominating ^1^H NMR spectroscopic features were that the 3-oxopropanol side chains with MTPA moiety were in equatorial position, and bend to C-13 position; CH_2_-1–O–C=O–CF_3_ substructure of Mosher esters of **1** were coplanar (Figure 4). The shielding or deshielding effects of the phenyl rings of MTPA moiety on H-13 or H-14 were larger in its *R*-Mosher ester than that of *S*-Mosher ester. The absolute configuration of C-13 in **1** was deduced as *S*-configuration based on the positive chemical shift differences (Δδ*^SR^*) of H-13 and negative chemical shift differences (Δδ*^SR^*) of H-14 from corresponding Mosher esters [6,7]. Finally, the absolute configuration of **1** was confirmed as (4*S*,5*S*,8*S*,9*R*,10*R*,13*S*)-configuration (Figure 1) based. The absolute configuration of **1** was validated by the result that the experimental data and calculated ECD spectrum for (4*S*,5*S*,8*S*,9*R*,10*R*,13*S*)-configuration of **1** matched exactly (Figure 5).

**Table 1:** ^1^H and ^13^C NMR data of compounds **1**–**5** (400/100 MHz in CDCl_3_, *J* in Hz).

	**1 ^α^**		**2**		**3**		**4**		**5**	
	^13^C	^1^H	^13^C	^1^H	^13^C	^1^H	^13^C	^1^H	^13^C	^1^H
1	58.0 t	3.82 m	57.9 t	3.80 m	58.2 t	3.82 m	58.4 t	3.82 m	61.2 t	4.08 dd 12.0, 8.0
										3.87 dt 12.0, 2.8
2	41.0 t	2.64 ddd 18.6, 6.6, 4.2	41.0 t overlapped	2.68 ddd 18.4, 6.0, 4.8	40.9 t	2.66 q 4.4	44.1 t	3.11 ddd 18.8, 6.4, 3.6	39.5 t	2.79 ddd 14.0, 12.8, 8.0
		2.63 ddd 18.6, 6.6, 4.2		2.61 ddd 18.4, 6.0, 4.8				2.67 ddd 18.8, 6.4, 3.6		2.19 ddd 14.0, 12.8, 8.0
3	215.4 s		215.6 s		215.8 s		216.1 s		212.6 s	
4	52.3 s		52.3 s		52.4 s		57.2 s		57.1 s	
5	45.3 d	1.66 m	39.0 d	1.59 m	38.7 d	1.58 m	43.3 d	1.78 m	43.0 d	2.23 m
6	26.8 t	1.54 m	26.7 t	1.68 m	23.0 t	1.53 m	23.1 t	1.42 m	25.8 t	1.14 m
		0.91 m		0.91 m		1.26 m		1.31 m		
7	33.5 t	1.16 m,	29.8 t	1.80 m	45.8 t	1.71 m	39.5 t	1.67 m	29.6 t	1.83 m
		1.71 m		1.08 m		1.27 m		1.50 m		1.02 m
8	41.0 d	1.36 m	41.0 d overlapped	1.58 m	70.2 s		70.1 s		40.8 d	1.62 m
9	79.3 d	2.89 t 9.6	36.3 t	1.84 m	39.7 t	1.65 m	45.3 t	1.74 m	35.4 t	1.94 m
				0.86 m		1.53 m		1.25 m		1.03 m
10	36.6 d	1.67 m	37.9 d	1.69 m	33.6 d	2.13 m	33.7 d	2.24 tt 11.8, 2.8	37.4 d	1.82 m
11	125.0 d	5.91 d 10.6	129.6 d	5.32 d 10.0	129.6 d	5.32 d 9.6	131.0 d	5.34, s	134.3 d	5.66 dd 9.6, 1.2
12	130.6 d	5.58 ddd 10.6, 4.8, 2.4	129.7 d	5.45 ddd 10.0, 4.8, 2.4	130.0 d	5.52 ddd 9.6, 4.8, 2.8	133.6 d	5.34, s	130.6 d	5.52 dd 9.6, 2.8
13	39.5 d	2.01 m	39.9 d	2.06 m	40.0 d	2.09 m	74.0 s		78.5 s	
14	18.6 q	0.75 d 8.4	18.7 q	0.72 d 7.2	18.8 q	0.75 d 7.2	27.5 q	1.13 s	20.5 q	1.18 s
15	17.5 q	1.19 s	17.4 q	1.17 s	17.7 q	1.22 s	12.1 q	1.33 s	11.2 q	0.88 s
16	18.7 q	1.00 d 9.6	68.3 t	3.44 dd 10.8, 6.4	31.8 q	1.22 s	31.8 q	1.25 s	68.3 t	3.48 m
				3.41 dd 10.8, 6.4						

The data were recorded at ^α^ 600 MHz (^1^H-NMR) and 150 MHz (^13^C-NMR).

Peaurantiogriseol B (**2**, Figure 1) was obtained as a white solid and had a molecular formula of C_16_H_26_O_3_ based on HREIMS data (observed *m*/*z* 266.1875 M^+^, calculated 266.1876), same as compound **1**. The ^1^H- and ^13^C-NMR spectra of **2** were very similar to those of **1** (Table 1), except for the absence of one oxygenated CH-9 group signal, and the change of a doublet signal at δ 1.00/18.7 to hydroxymethyl signals at δ 3.44/3.41/68.3. These results suggested the presence on compound **2** of a hydroxymethyl group at C-16 position. The ^1^H-^1^H COSY and HMBC correlations of **2** were also similar to those of **1** (Figure 2), which confirmed that an OH group was located at C-16.

The relative stereochemistry of **2** was established by its NOESY spectrum (Figure 3). Its NOE data were very similar to those of **1**. A *trans*-fused decalin ring in **2** was indicated by the correlations of H-15/H-13, H-15/H-10, and H-14/H-5. Methyl-15 and methyl-14 were oriented on the opposite side, and hydroxymethyl-16 and methyl-14 were oriented on the same side based on the NOE correlation of H-16/H-5.

The absolute configuration of **2** was determined by comparison of the ^1^H-NMR spectra of its (*R*) and (*S*)-Mosher esters like compound **1**. Both alcohols (OH-1 and OH-16) in **2** were esterified based on the larger chemical shifts of H-1/H-16 (Supplementary Materials Table S2), which were confirmed based on its ^19^F NMR spectra (Supplementary Materials, Figure 13S); there were 3 CF_3_ signals in ^19^F NMR spectra of Mosher esters of **2**. The ^1^H NMR spectroscopic features and preferred conformations of Mosher esters of **2** were the same as that of **1**. Therefore, the absolute configuration of **2** was confirmed as (4*S*,5*S*,8*S*,10*R*,13*S*)-configuration (Figure 1). The absolute configuration of **2** was validated by the result that the experimental data and calculated ECD spectrum for (4*S*, 5*S*, 8*S*, 10*R*, 13*S*)-configuration of **2** matched exactly (Figure 5).

Peaurantiogriseol C (**3**, Figure 1) was obtained as a white solid and had a molecular formula of C_16_H_26_O_3_ based on HREIMS data (observed *m*/*z* 248.1770 [M − H_2_O]^+^, calculated for C_16_H_24_O_2_, 248.1771). The ^1^H- and ^13^C-NMR spectra of **3** were very similar to those of compound **2** (Table 1), except for the changes of hydroxymethyl signals at δ 3.41/68.3 to a methyl singlet at δ 1.22/31.0 in **3**, and CH group signals at δ 1.58/41.0 to quaternary carbon atom signal at δ 70.2 in **3**. These results suggested that compound **3** possesses a hydroxyl group at C-8 position, which were confirmed by the HMBC correlations from H-16 to C-7, and H-16 to C-9 (Figure 2). The relative stereochemistry of **3** was the same as compound **2** based of its NOESY spectrum (Figure 3). The absolute configuration of compound **3** was determined by the result that the experimental data, showing a negative Cotton effect at 291 nm, and calculated ECD spectrum for (4*S*,5*S*,8*S*,10*R*,13*S*)-configuration of **3** matched exactly (Figure 5).

Peaurantiogriseol D (**4**, Figure 1) had a molecular formula of C_16_H_26_O_4_ based on HREIMS data (observed *m*/*z* 282.1824 M^+^, calculated 282.1826), with one more oxygen atom than compound **3**. The ^1^H-NMR, ^13^C-NMR, ^1^H–^1^H COSY, and HMBC correlations of **4** were very similar to those of **3** (Table 1, Figure 2), except for the change of a methyl doublet signal at δ 0.75 (d, *J* = 7.2 Hz) to singlet signal at δ 1.13 in **4**, which suggested that compound **4** had an added OH group. The additional OH group was located at C-13 based on the chemical shift of CH-13 (δ_c_ 74.0) and HMBC correlations. The relative stereochemistry of **4** was established based on the result that the interatomic non-bonded distance of key atoms and NOESY correlation signals matched exactly in its 3D model (Table 2, Me-eea-*trans* conformer). The relative stereochemistry of **4** was assigned as that methyl-14 and methyl-15 were equatorial; methyl-16 was axial; and the decalin ring was *trans* based on the interatomic non-bonded distance of key atoms less than 4 Å. The *trans*-fused decalin ring of **4** was supported by the coupling constant of CH-10 signal (tt, *J* = 11.8, 2.8 Hz). The absolute configuration of **4** was confirmed as (4*R*,5*S*,8*S*,10*R*,13*R*)-conformer by the result that the experimental data, showing a negative Cotton effect at 298 nm, and calculated ECD spectrum for (4*R*,5*S*,8*S*,10*R*,13*R*)-conformer of **4** matched exactly (Figure 5).

**Table 2:** The key NOE correlations of compound **4** and interatomic non-bonded distance of the key atoms in its main 3D conformers.

Key Atoms	NOE Correlation	Main 3D Conformers		
		Me-aaa-*Cis*	Me-eee-*Cis*	Me-eea-*Trans*
		Distance (Å)	Distance(Å)	Distance(Å)
CH_3_-14	H-5	3.347715	4.256464	3.471439
CH_3_-15	H-10	3.608014	2.503497	1.862233
CH_3_-16	H-10	2.310649	5.322337	1.826166
CH_3_-14	H-2	5.109479	4.964076	2.470492
CH-5	H-10	2.265362	2.503497	3.087856

Me-aaa-*cis* conformer: methyl-14, methyl-15, and methyl-16 are axial, decalin ring is *cis*, hypothetically; Me-eee-*cis* conformer: methyl-14, methyl-15, and methyl-16 are equatorial, decalin ring is *cis*, hypothetically; Me-eea-*trans* conformer: methyl-14 and methyl-15 are equatorial, and methyl-16 is axial, decalin ring is *trans*, hypothetically.

Peaurantiogriseol E (**5**, Figure 1) had a molecular formula of C_16_H_24_O_3_ based on HREIMS data (observed *m*/*z* 264.1721 M^+^, calculated 264.1720), which was two mass units less than that of compound **2**, requiring 5 degrees of unsaturation. The ^1^H-NMR and ^13^C-NMR data for **5** were similar to those of compound **2** (Table 1). The most obvious difference between **5** and **2** was that the absence of one CH group signal at δ 2.06/39.9, and the change of a doublet signal at δ 0.72 to singlet at δ 1.18/20.5 in **5**. These results suggested that a pyrone moiety was formed in **5** by an O–C bond at C-13 position, which was supported by the HMBC correlations from H-1 to C-13 (Figure 2), and, from H-2 to C-4.

The relative stereochemistry of **5** was established by its NOESY spectrum (Figure 3). A *cis*-fused decalin ring in **5** was confirmed based on the NOE correlations of H-15/H-10, H-14/H-5, and H-15/H-14. Methyl-14, methyl-15, and hydroxymethyl-16 were oriented on the same side by the NOE correlations between H-16 and H-10. The absolute configuration of **5** was determined based on the result that the experimental ECD spectrum, showing a negative Cotton effect at 298 nm, and calculated ECD spectrum for (4*R*,5*R*,8*R*,10*R*,13*S*)-configuration of compound **5** matched exactly (Figure 5).

Peaurantiogriseol F (**6**, Figure 1) had a molecular formula of C_16_H_26_O_4_ based on HRESIMS data (observed *m*/*z* 283.18999 [M + H]^+^, calculated for C_16_H_26_O_4_, 283.19039 [M + H]^+^), with the same planar structure as that of known craterellone D based on its spectroscopic data (Supplementary Materials Table S1) [8]. The ${\left[\alpha \right]}_{\text{D}}^{25}$ of compound **6** was +21 (*c* 2.2, MeOH) with opposite sign of craterellone D. The NOE correlations of H-14/H-15, H-14/H-5, H-14/H-10, H-15/H-5, and H-15/H-10 allowed us to unambiguously establish a *cis-*fused decalin ring in **6** different from the *trans* junction of craterellone D. Hydroxymethyl-16 were oriented on the opposite side of methyl-14/methyl-15 based on the NOE correlation of H-8/H-10. Compound **6** is a diastereoisomer of craterellone D. The absolute configuration of **6** was determined based on the result that the experimental ECD spectrum, showing a negative Cotton effect at 296 nm, and calculated ECD spectrum for (4*S*,5*S*,8*R*,10*S*,13*R*)-configuration of **6** matched exactly (Figure 5).

Compound **7** was identified as aspermytin A by comparison of its spectral data with [9]; both compound **7** and aspermytin A had the same NMR, MS and specific rotation data.

The structure of compound **8** (Figure 1) was also elucidated by its spectroscopic data (Supplementary Materials Table S1). Its ${\left[\alpha \right]}_{\text{D}}^{25}$ was −29 (*c* 2.1, MeOH). Its *trans-*fused decalin ring was determined based on the NOE correlations of H-14/H-5 and H-15/H-10; its methyl-14 and hydroxyl-9 were oriented on the opposite side of methyl-15 and methyl-16 by the NOE correlations of H-15/H-10, H-16/H-10, and H-16/H-9. It was found that the planar structure of compound **8** was the same as that of known 1-propanone, 3-hydroxy-1-(1,2,4a,5,6,7,8,8a-octahydro-2,5-dihydroxy-1,2,6-trimethyl-1-naphthalenyl), CAS Registry Number 1235005-17-0. No reference, nor stereochemical information are presented in SciFinder.

Compounds **1**−**8**, at a concentration of 50 mM, showed low inhibitory effect against human aldose reductase; the corresponding value of percent inhibition were 16%, 6%, 31%, 22%, 26%, 2%, 13%, and 9%. The compounds showed no activity of inducing neurite outgrowth (PC-12) [9], nor antimicrobial activity against *E. coli.* (ATCC 25922), *Staphylococcu aureus* (ATCC 25923), and *Candida albicans* (ATCC 60193), at a concentration of 128 μg/mL.

## 3. Experimental Section

### 3.1. General Experimental Procedures

(*R*)-(−)-α-Methoxy-α-trifluoromethylphenylacetyl chloride (MTPA-Cl) (99%), (*S*)-(+)-MTPA-Cl (99%), dimethyl sulfoxide, and pyridine-*d*_5_ (99.5%) were purchased from Sigma (St. Louis, MO, USA). 6×His-tagged recombinant human aldose reductase was presented by Xiaopeng Hu, School of Pharmaceutical Sciences, Sun Yat-sen University [10]; its substrate, β-NADPH were purchased from Sigma-Aldrich (St. Louis, MO, USA); methanol was HPLC grade; other reagents were analytical grade and commercially available; PDA medium were purchased from Beijing Land Bridge Technology Co., Ltd, Beijing, China.

Optical rotation measurements were carried out using a Bellingham-Stanley 37–440 polarimeter (Bellingham Stanley Ltd., Kent, UK). UV spectra were determined using a UV-240 spectrophotometer (Shimadzu, Tokyo, Japan). ECD spectra were measured using a Chirascan Circular Dichroism Spectrometer (Applied PhotoPhysics, Surrey, UK). IR spectra were measured on a TENSOR37 spectrometer (Bruker Optics, Ettlingen, Germany). The ^1^H-NMR and ^13^C-NMR data were acquired using a Bruker Avance 400 spectrometer at 400 MHz for ^1^H nuclei and 100 MHz for ^13^C nuclei, a Bruker Avance III 500 MHz NMR spectrometer at 470 MHz for ^19^F nuclei, and a Bruker Avance III 600 MHz NMR spectrometer at 600 MHz for ^1^H nuclei and 150 MHz for ^13^C nuclei (Bruker Biospin, Rheinstetten, German). TMS was used as an internal standard, and the chemical shifts (δ) were expressed in ppm or Hz. The EI mass spectra and high-resolution mass spectra were obtained using MAT95XP (ThermoFinnigan, Bremen, Germany) high resolution mass spectrometer and a LTQ-Orbitrap LC-MS (Thermo Fisher, Frankfurt, German). HPLC was performed using a 515 pump with a UV 2487 detector (Waters, Milford, MA, USA) and an Ultimate XB-C-18 column (250 mm × 10 mm, 5 μm; Welch, MD, USA). Normal pressure preparative column chromatography was carried out on RP-18 gel (25–40 μm, Daiso Inc., Osaka, Japan), silica gel (200–400 mesh, Qingdao Marine Chemical Inc., Qingdao, China), or Sephadex-LH-20 (GE Healthcare, Stockholm, Sweden) for reverse and direct phase elution modes, respectively. TLC was performed over F_254_ glass plates (Qingdao Marine Chemical Inc., Qingdao, China) and analyzed under UV light (254 and 366 nm).

### 3.2. Fungal Material

Endophytic fungus *Penicillium aurantiogriseum* 328# was isolated with PDA medium from the bark of *Hibiscus tiliaceus* collected in the Qi’ao Mangrove Nature Reserve of Guangdong Province, China and identified according to its morphological characteristics and the ITS region [11]. A voucher specimen is deposited in our laboratory at −20 °C.

### 3.3. Fermentation, Extraction and Isolation

Small agar slices bearing mycelia were placed in 1000 mL Erlenmeyer flasks containing rice medium (composed of 60 g rice, 80 mL distilled water, and 0.24 g sea salt); and incubated for 30 days at 28 °C. In total, 120 flasks of culture were obtained. Cultures were extracted with EtOAc. In total, 200 g crude extract was obtained by evaporation of EtOAc. The crude extract was suspended in H_2_O (2 L) and partitioned with *n*-hexane (3 L × 2) and EtOAc (3 L × 2) to give *n*-hexane (90 g) and EtOAc (51 g) extracts, respectively.

The EtOAc extract was subjected to silica gel column, eluted with *n*-hexane–EtOAc gradient (from 100:0 to 0:100) to obtain six fractions (Frs. 1–6). Fr. 2 (7.5 g) was subjected to column chromatography over silica gel, eluted with *n*-hexane–EtOAc (50:50) to obtain three fractions (Frs. 2.1–2.3). Frs. 2.1 and Frs. 2.2 were purified by Sephadex LH-20 (MeOH) to yield compound **1** (67.5 mg) and compound **7** (1.007 g), respectively; Fr. 2.3 was separated by HPLC (MeOH-H_2_O, 20:80, 2 mL/min, 254 nm) to isolate compound **5** (6 mg). Fr. 3 (4.5 g) was purified using a Sephadex LH-20 column (MeOH) and separated by HPLC (MeOH-H_2_O gradient from: 25:75 to 50:50, 2 mL/min, 254 nm) to yield compound **3** (40 mg) and compound **2** (45 mg). Fr. 4 (7.7g) was subjected to column chromatography over silica gel, eluted with *n*-hexane–EtOAc (40:69) to obtain two fractions, Fr. 5.1 and Fr. 5.2. Fr. 5.1 purified using Sephadex LH-20 (MeOH) to yield compound **6** (206 mg); and Fr. 5.2 was separated by HPLC (MeOH-H_2_O, 20:80, 2 mL/min, 254 nm) to yield compound **4** (4 mg) and compound **8** (27.1 mg).

### 3.4. Spectral Data

Peaurantiogriseol A (**1**): colorless solid; ${\left[\alpha \right]}_{\text{D}}^{25}$ +73 (*c* 1.5, MeOH); UV (MeOH) λ_max_ (logε) 240 (2.4) nm; ECD (MeOH) Δε_249_ −0.36; IR (KBr) ν_max_ 3425, 1703 cm^−1^; for ^1^H-NMR and ^13^C-NMR data, see Table 1; EIMS *m*/*z* 266 (M^+^), 248, 217, 203, 193; HREIMS *m*/*z* 266.1878 [M^+^] (calculated for C_16_H_26_O_3_, 266.1876).

Peaurantiogriseol B (**2**): colorless solid; ${\left[\alpha \right]}_{\text{D}}^{25}$ +89 (*c* 1.7, MeOH); UV (MeOH) λ_max_ (logε) 240 (3.1) nm; ECD (MeOH) Δε_249_ −0.49; IR (KBr) ν_max_ 3431, 1701 cm^−1^; for ^1^H-NMR and ^13^C-NMR data, see Table 1; EIMS *m*/*z* 266 [M^+^]; HREIMS *m*/*z* 266.1875 [M^+^] (calculated for C_16_H_26_O_3_, 266.1876).

Peaurantiogriseol C (**3**): colorless solid; ${\left[\alpha \right]}_{\text{D}}^{25}$ +60 (*c* 0.5, MeOH); UV (MeOH) λ_max_ (logε) 250 (2.1) nm; ECD (MeOH) Δε_291_ −0.21; IR (KBr) ν_max_ 3434, 1701 cm^−1^; for ^1^H-NMR and ^13^C-NMR data, see Table 1; EIMS *m*/*z* 248 (M-H_2_O)^+^, 206, 175; HREIMS *m*/*z* 248.1770 [M − H_2_O]^+^ (calculated for C_16_H_24_O_2_, 248.1771).

Peaurantiogriseol D (**4**): white solid; ${\left[\alpha \right]}_{\text{D}}^{25}$ +18 (*c* 0.4, MeOH); UV (MeOH) λ_max_ (logε) 282 (2.6) nm; ECD (MeOH) Δε_298_ −0.88; IR (KBr) ν_max_ 3434, 1688 cm^−1^; for ^1^H-NMR and ^13^C-NMR data, see Table 1; EIMS *m*/*z* 282 [M^+^]; HREIMS *m*/*z* 282.1824 [M^+^] (calculated for C_16_H_26_O_4_, 282.1826).

Peaurantiogriseol E (**5**): colorless solid; ${\left[\alpha \right]}_{\text{D}}^{25}$ −153 (*c* 1.5, MeOH); UV (MeOH) λ_max_ (logε) 282 (2.0) nm; ECD (MeOH) Δε_298_ −2.5; IR (KBr) ν_max_ 3470, 1686 cm^−1^; for ^1^H-NMR and ^13^C-NMR data, see Table 1; EIMS *m*/*z* 264 [M^+^], 249, 231, 192; HREIMS *m*/*z* 264.1721 [M^+^] (calculated for C_16_H_24_O_3_, 264.1720).

Peaurantiogriseol F (**6**): white solid; ${\left[\alpha \right]}_{\text{D}}^{25}$ +21 (*c* 2.2, MeOH); UV (MeOH) λ_max_ (logε) 289 (1.9) nm; ECD (MeOH) Δε_296_ −1.61; for ^1^H-NMR and ^13^C-NMR data, see Supplementary Materials table S1; EIMS *m*/*z* 282 (M^+^), 264, 249, 203, 173; HRESIMS *m*/*z* 283.18999 [M + H]^+^ (calculated for C_16_H_27_O_4_, 283.19039 [M + H]^+^).

### 3.5. Computational Analyses

All the theoretical methods and the basis set used for optimization and spectrum calculation were recommended in previous studies [12,13]. All the theoretical calculations, including geometry optimization, frequency analysis, and ECD spectrum prediction, were carried out with the density functional theory (DFT) and time-dependent density functional theory (TDDFT) methods in the Gaussian 09 software package [14]. The geometry optimizations were performed at the B3LYP/6-31+G (d) level in the gas phase. Based on the final optimized structure, the ECD spectra were calculated at the PBE1PBE-SCRF/6-311++g (d, p) level using the PCM solvent continuum models with methanol as a solvent. The theoretical predicted ECD spectra were fitted in the SpecDis software package.

### 3.6. Esterification Procedure

The Mosher esters of compounds **1** and **2** were prepared by treatment of compounds **1** and **2** with corresponding (*R*)-(−)-α-methoxy-α-trifluoromethylphenylacetyl chloride (1.5 equivalence ratio), or (*S*)-(+)-α-methoxy-α-trifluoromethylphenylacetyl chloride (1.5 equivalence ratio) in pyridine-*d*_5_ under a nitrogen atmosphere. The reaction mixtures were stood at room temperature for 3.5 h. Then, ^1^H NMR spectra of the samples were recorded at 400 MHz.

### 3.7. Inhibition of Aldose Reductase

The method to examine the inhibition of aldose reductase was similar to the method used by Michael C. Van Zandt *et al.* [15]. Enzyme activity was measured by monitoring the rate of disappearance of NADPH at 340 nm. The reaction contents in a final volume of 300 μL were 6.6% *w*/*v* (NH_4_)_2_SO_4_, 33 mM NaH_2_PO4 (pH 6.6), 0.11 mM NADPH, 4.7 mM dl-glyceraldehyde, 0.59 μg of enzyme, 1% DMSO, and compound. Each assay was done in triplicate. Percent inhibition was calculated on the basis of enzyme activity in the presence or absence of compound.

## 4. Conclusions

Polyketide decalin-derived secondary metabolites are widely found in nature [16,17,18]; Abundant polyketide decalin derivatives (**1**–**8**) from endophytic fungus 328# from the mangrove in the South China Sea were reported in this work; their absolute configurations were established by theoretical calculations of electronic circular dichroism and NMR data analyses of Mosher ester derivatives. Compounds **1**–**8** showed low activity under our experimental conditions.

## Supplementary Files

Supplementary File 1
