# Supplementary material for: Six New Polyketide Decalin Compounds from Mangrove Endophytic Fungus Penicillium aurantiogriseum 328#
Source: Mar Drugs. 2015 Oct 10;13(10):6306–18. doi: 10.3390/md13106306 (PMC4626691; doi:10.3390/md13106306)
Supplement: Supplementary File 1 [file marinedrugs-13-06306-s001.docx]

Supplementary Materials





**Figure S1.** ^1^H NMR for peaurantiogriseol A (**1**).

**

**

**Figure S2.** ^13^C NMR for peaurantiogriseol A (**1**).





**Figure S3.** ^1^H−^1^H COSY for peaurantiogriseol A (**1**).





**Figure S4.** HSQC for peaurantiogriseol A (**1**).





**Figure S5.** HMBC for peaurantiogriseol A (**1**).





**Figure S6.** NOESY for peaurantiogriseol A (**1**).





**Figure S7.** ^1^H NMR for (*R*) and (*S*)-Mosher esters of peaurantiogriseol A (**1**).





**Figure S8.** ^19^F NMR spectra of Mosher esters of peaurantiogriseol A (**1**).
**A**/**B**: peaurantiogriseol A (**1**) esterified by *S*/*R*-MTPA-Cl.





**Figure S9.** ^1^H NMR for peaurantiogriseol B (**2**).





**Figure S10.** ^13^C NMR for peaurantiogriseol B (**2**).





**Figure S11.** NOESY for peaurantiogriseol B (**2**).





**Figure S12.** ^1^H NMR for (*R*) and (*S*)-Mosher esters of peaurantiogriseol B (**2**).





**Figure S13.** ^19^F NMR spectra of Mosher esters of peaurantiogriseol B (**2**). **A**/**B**: peaurantiogriseol B (**2**) esterified by *S/R*-MTPA-Cl.





**Figure S14.** ^1^H NMR for peaurantiogriseol C (**3**).


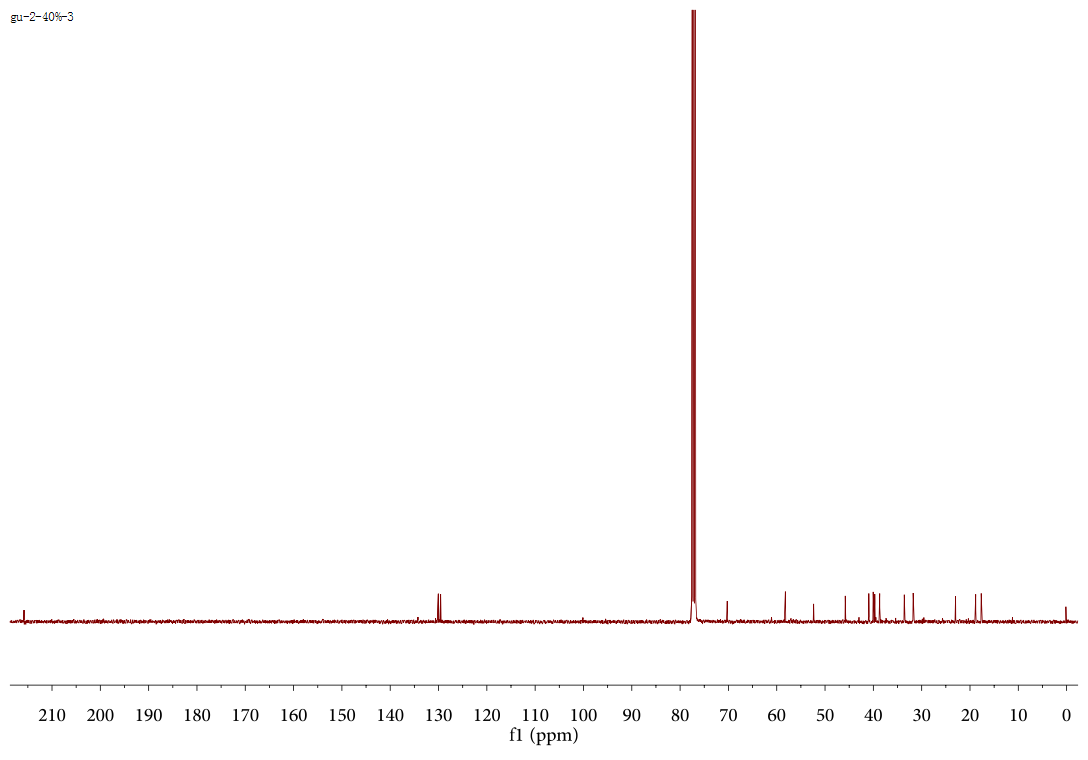


**Figure S15.** ^13^C NMR for peaurantiogriseol C (**3**).


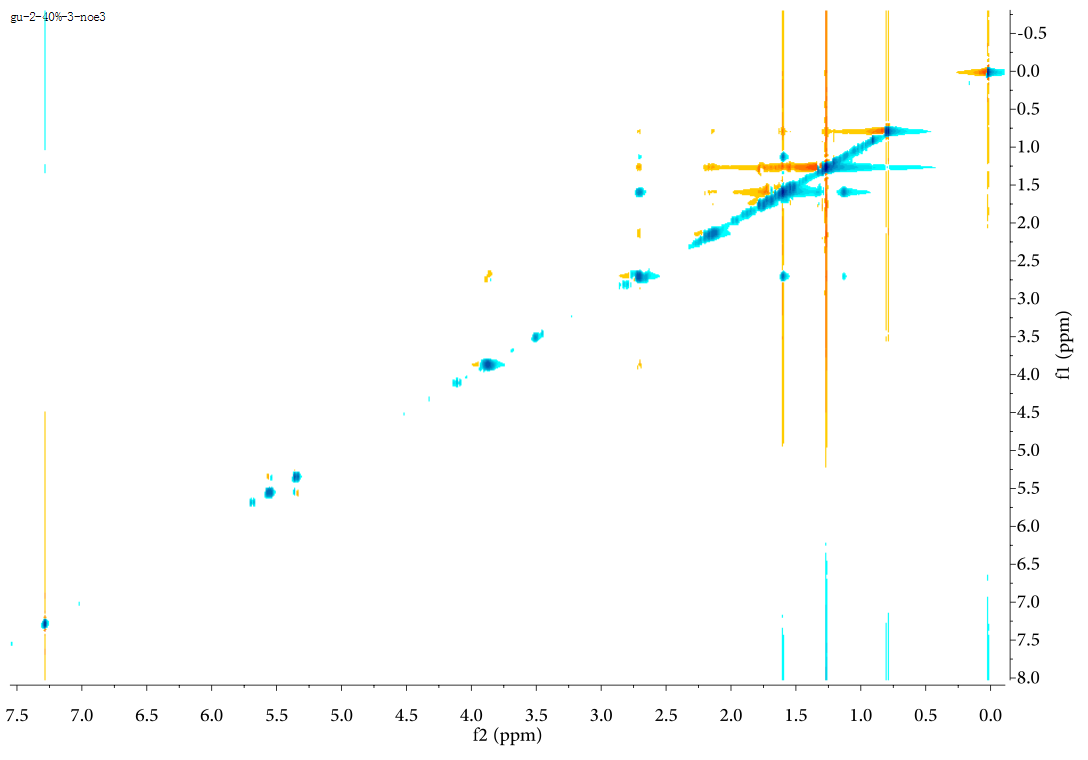


**Figure S16.** NOESY for peaurantiogriseol C (**3**).





**Figure S17.** ^1^H NMR for peaurantiogriseol D (**4**).


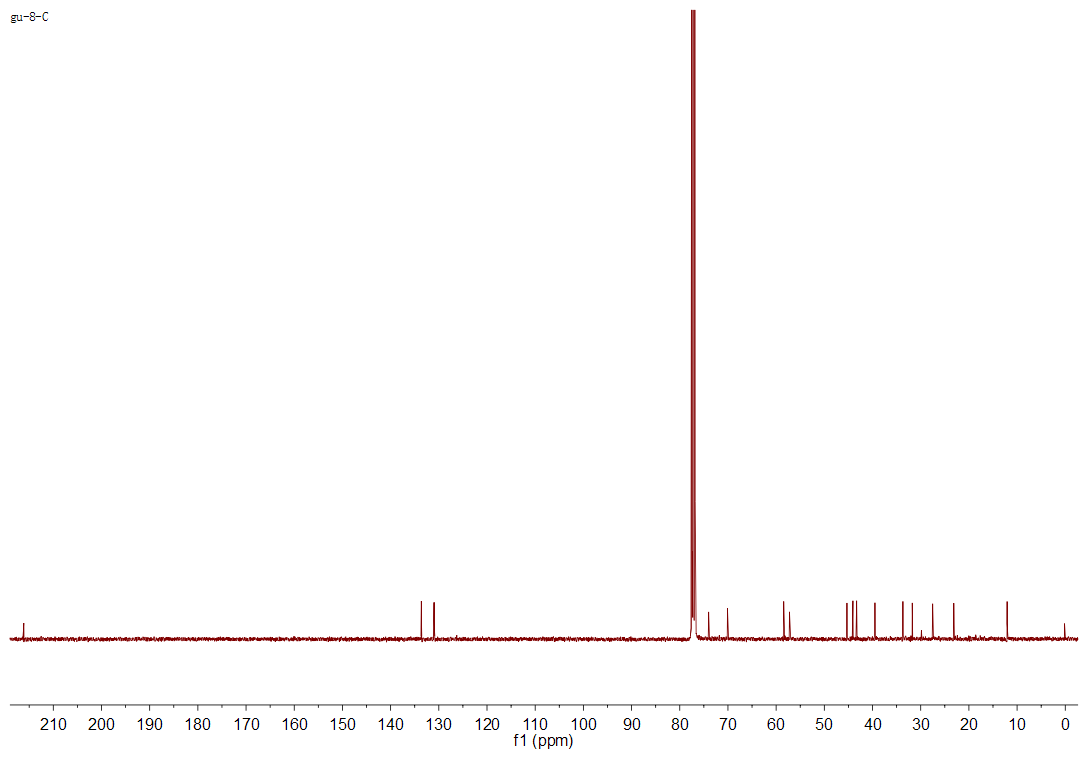


**Figure S18.** ^13^C NMR for peaurantiogriseol D (**4**).





**Figure S19.** NOESY for peaurantiogriseol D (**4**).





**Figure S20.** ^1^H NMR for peaurantiogriseol E (**5**).





**Figure S21.** ^13^C NMR for peaurantiogriseol E (**5**).





**Figure S22.** NOESY for peaurantiogriseol E (**5**).


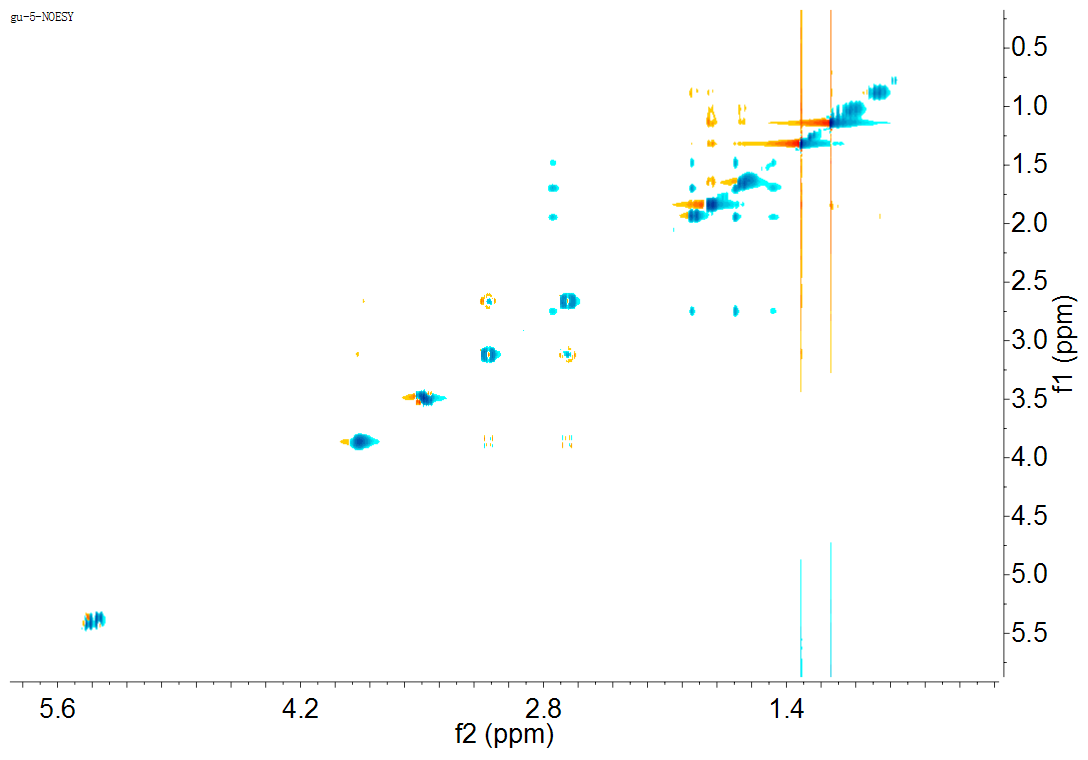


**Figure S23.** NOESY for peaurantiogriseol F (**6**).





**Figure S24.** ECD spectra for peaurantiogriseols A−F (**1**−**6**).

**Table S1.** NMR data of compounds **6** and **8** (400/100 MHz in CCl_3_D).

|  | **6** | | | **8** | | |
| --- | --- | --- | --- | --- | --- | --- |
|  | **^13^C** | **^1^H (Hz)** | **HMBC** | **^13^C** | **^1^H (Hz)** | **HMBC** |
| 1 | 58.4 t | 3.84 m | 2 | 58.4 t | 3.81 m | 2 |
| 2 | 44.1 t | 3.10 ddd 18.8, 6.4, 4.0 |  | 44.2 t | 3.08 dt 5.2, 18.8 | 1 |
|  |  | 2.64 ddd 18.8, 6.4, 4.0 |  |  | 2.60 m |  |
| 3 | 216.1 s |  | 2, 15 | 216.0 s |  | 1, 2, 15 |
| 4 | 57.3 s |  | 12, 14, 15 | 57.3 s |  | 12, 5, 15, 14 |
| 5 | 38.1 d |  | 11, 10, 7 | 41.3 d | 1.87 m | 11, 10, 15 |
| 6 | 29.6 t | 1.83 m |  | 27.0 t | 1.46 m |  |
|  |  | 1.83 m |  |  | 0.99 m |  |
| 7 | 27.0 t | 1.07 m | 6 | 33.4 t | 1.71 m | 16 |
|  |  | 1.63 m |  |  | 1.14 m |  |
| 8 | 41.0 d | 1.00 m | 16, 9 | 41.1 d | 1.34 m | 7, 16 |
| 9 | 35.8 t | 1.64 m | 11, 16 | 79.0 d | 2.85 m | 11, 10, 7, 16 |
|  |  | 1.91 m |  |  |  |  |
| 10 | 43.7 d | 0.86 m | 12, 9 | 45.4 d | 1.71 m | 11, 12, 5 |
| 11 | 133.4 d | 1.82 m | 14 | 126.4 d | 5.94 d 10.0 | 10 |
| 12 | 130.9 d | 5.34 dd 10.0, 2.0 |  | 134.3 d | 5.40 dt 10.0, 2.0 | 10, 14 |
| 13 | 74.0 s | 5.40 d 10.0 | 11, 12, 14, 15 | 73.5 s |  | 11, 12, 5, 15, 14 |
| 14 | 27.5 q |  |  | 27.5 q | 1.09 s | 12 |
| 15 | 12.0 q | 1.12 s |  | 12.0 q | 1.27 s | 5 |
| 16 | 68.4 t | 1.29 s | 7, 8 | 18.7 q | 1.00 d 6.7 | 10, 7 |
|  |  | 3.47 m |  |  |  |  |

**Table S2.** ^1^H NMR data and Δ*δ^SR^* values of *S*/*R*-MTPA esters of **1** and **2** (400 MHz in pyridine-*d*_3_).

|  | **1** | | | | **2** | | | |
| --- | --- | --- | --- | --- | --- | --- | --- | --- |
|  | **δ_H_ (ppm)** | | **Δδ^SR^(=δ^S^ − δ^R^)** | | **δ_H_ (ppm)** | | **Δδ*^SR^*(=δ^S^ − δ^R^)** | |
|  | ***S-*MTPA Ester** | ***R-*MTPA Ester** | **ppm** | **Hz** | ***S-*MTPA Ester** | ***R-*MTPA Ester** | **ppm** | **Hz** |
| 1a | 4.82 | 4.87 | −0.05 | −20 | 4.81 | 4.89 | −0.08 | −32 |
| 1b |  | 4.74 | 0.08 | 32 |  | 4.73 | 0.08 | 32 |
| 2 | 2.98 | 2.94 | 0.04 | 16 | 2.96 | 2.91 | 0.04 | 16 |
| 5 | 1.72 | 1.72 | 0.00 | 0 | 1.59 | 1.59 | 0.00 | 0 |
| 6 | 1.60 | 1.60 | 0.00 | 0 | 0.90 | 0.90 | 0.00 | 0 |
| 7 | 1.16 | 1.16 | 0.00 | 0 | 1.70 | 1.70 | 0.00 | 0 |
| 8 | 1.36 | 1.36 | 0.00 | 0 | 1.58 | 1.58 | 0.00 | 0 |
| 9 | 3.11 | 3.12 | 0.01 | 4 | 1.70 | 1.70 | 0.00 | 0 |
| 10 | 1.97 | 1.98 | −0.02 | −8 | 1.73 | 1.73 | 0.00 | 0 |
| 11 | 6.52 | 6.52 | 0.00 | 0 | 5.30 | 5.30 | 0.00 | 0 |
| 12 | 5.68 | 5.66 | 0.02 | 8 | 5.53 | 5.51 | 0.02 | 8 |
| 13 | 2.05 | 2.00 | 0.05 | 20 | 2.05 | 1.99 | 0.06 | 24 |
| 14 | 0.75 | 0.79 | −0.04 | −16 | 0.70 | 0.74 | −0.04 | −16 |
| 15 | 1.19 | 1.19 | 0.00 | 0 | 1.13 | 1.12 | 0.01 | 4 |
| 16 | 1.24 | 1.24 | 0.00 | 0 | 4.25 | 4.27 | 0.02 | 8 |
|  |  |  |  |  | 4.18 | 1.19 | 0.01 | 4 |

© 2015 by the authors; licensee MDPI, Basel, Switzerland. This article is an open access article distributed under the terms and conditions of the Creative Commons Attribution license (http://creativecommons.org/licenses/by/4.0/).
